# The Antagonism between Opium and Bella-Donna

**Published:** 1892-12

**Authors:** David Cerna

**Affiliations:** Demonstrator of Physiology to the Medical Department of the University of Texas; formerly Demonstrator of, and Lecturer on, Experimental Therapeutics in the Univerity of Pennsylvania, etc.


### DANIEL'S TEXAS MEDICAL JOURNAL.

ESTABLISHED JULY, 1885.

PUBLISHED MONTHLY.—SUBSCRIPTION $2.00 A YEAR.

Vol. VIII. AUSTIN, DECEMBER, 1892. No. 6.

### Original Contributions.

For Daniel's Texas Medical Journal.

### THE ANTAGONISM BETWEEN OPIUM AND BELLADONNA.

BY DAVID CERNA, M. D., PH. D.,

Demonstrator of Physiology to the Medical Department of the University of Texas; formerly Demonstrator of, and Lecturer on, Experimental Therapeutics in the Univerity of Pennsylvania, etc.

[Read before the Galveston County Medical Society at Galveston, November 21st, 1892.]

I SHALL avail myself of this opportunity to bring before the Society a subject of the greatest interest, not only from a scientiffc, but also from a practical point of view. Chiefly for the sake of brevity I shall avoid all unnecessary bibliographical references.

The antagonism alleged to exist between opium and belladonna has long been and still is a mooted question. In carefully examining the quite extensive literature of the subject, we come across a large mass of evidence which is mainly contradictory. In order, however, to better understand and study the matter, permit me to consider it under the chief two points of view, the experimental and the clinical.

THE ACTIONS OF OPIUM.

What are the predominant actions of opium or its chief alkaloid, morphine? I will confine myself to an examination of the actions exercised on the Circulation and the Respiration.

ON THE CIRCULATION.—THE HEART.

All observers agree more or less in that under opium, there is, at first, a slight acceleration of cardiac action, followed by the more constant effects of the drug, which are a slow, full and strong pulse. This returns, in the course of time, to the normal rate, or perhaps to an increased rapidity. Toxic doses, on the other hand, at first slow the pulse and then, through a depressant action, this becomes rapid and feeble. It has been shown experimentally that such phenomena are due, successively, to an evanescent stimulation of the heart muscle, and to an action of the drug on the cardiac-inhibitory centres, both |centrally and peripherally, followed by paralysis of the same. In other words, the first acceleration of the pulse is the result of a slight primary stimulation of the cardiac muscular fibre; the secondary diminution in rate to the excitation of the peripheral and central cardiac-inhibitory nerve apparatus; and the final rapid and feeble pulse to paralysis of these centres peripherally.

ON THE BLOOD-PRESSURE.

What influence opium exerts upon the arterial pressure is as yet not very well established. There is undoubtedly a slight primary rise which is soon followed by a condition of depression. How these phenomena are brought about has not been made out definitely. Some experimenters affirm that the vaso-motor system plays an important part in the manifestation of these changes. Yet, the evidence that we have is insufficient to sustain such an assertion. Though the primary rise of the blood-pressure may be attributed to an action of opium on the vaso-motor centres, the depression that follows is not wholly of voso-motor origin. Electrical stimulation of the spinal cord, at a time when the column of mercury in the manometer is far below the normal height, is almost immediately followed by a rise of the arterial pressure. The results of this experiment alone seem to show that the vaso-motor paralizant action claimed for the drug by some observers is purely imaginary. This point, however, needs further investigti o n.

ON THE RESPIRATION.

The general concensus of opinion in regard to the action of opium on the respiration, is that the agent is a decided depressant of this function. These effects are produced by the drug on normal animals as well as in those whose pneumogastrics have been previously divided. It is logically inferred from this, that the medicament exercises a direct action on the respiratory centres in the medulla oblongata. Most of the results of experimentation seem to point to the truth of this statement; and yet, I have seen not once but on several occasions, morphine producing an extraordinary increase in the number of the respiratory movements, even when large doses have been ingested. I have made these observations in the case of dogs. Such results as these that seem to overthrow the generally accepted recognition of opium as a respiratory depressant, I am at a loss to understand, and much less to explain in the present unsettled state of our imperfect knowledge of the whole subject of respiration, unless it be by idiosyncrasies in the lower animals, which must occur as they do in man. Nevertheless it is a well established fact that in the case of man, at least, opium depresses the respiratory function, and finally kills by respiratory failure through a direct action on the centres.

Let us now turn our thoughts to the

### ACTIONS OF BELLADONNA:

As in the case of opium, I will only dwell on the actions of this drug on the Circulation and the Respiration.

### ON THE CIRCULATION.—THE HEART.

It is generally held that belladonna, or its alkaloid atropine, produces an acceleration of the pulse, sometimes preceded by a decrease in rate. Such an effect is observed, at least, in man and for the greater part in the lower animals. This rapidity of the pulse is attributed to a probable stimulation of the accelerator nerves of the heart, and to a peripheral paralysis of the cardio-inhibitory nerve apparatus. Bnt the evidence is somewhat contradictory. Thus, in the heart of the frog, isolated or *in situ*, atropine, according to some observers, causes an increase in the number of pulsations, the contrary having been noticed by others. I, myself, have never seen the alkaloid, locally applied in solulion or hypodermatically injected, produce a rapid cardiac action in the batrachian, but always a decrease from the onset. In the case of mammals, as the dog, for instance, the action of the drug is uncertain, and I am inclined to accept, supported by my own observations, the results embodied in the recent most excellent and thorough research of Prof. Reichert. (*University Medical Magazine*, February, 1891.) There is, in fact, no evidence, much less proof, of the accelerator centres (or nerves, if you choose) being influenced by atropine in order to cause a rapid pulse. This action of the drug, though by no means constant, is observed in animals whose vagi have been previously severed, and similarly upon those in which the heart has been isolated from all nervous connection by section of both vagi and spinal cord, as well as in the normal animals. If atropine acted chiefly on the accelerator centres and nerves we would not expect to find a rapid pulse as often in the operated animals as in the normal animals. Small doses of the drug cause a rapid pulse in the isolated heart of the mammal, even when electrical stimulation of the pneumogastrics shows these to be intact. The same phenomina, and probably in a more marked degree, is observed under toxic quantities of the alkaloid, at a time when faradization of the vagi fails to influence the activity of the heart, thus showing that the drug has by this time brought about a paralysis, peripherally, of the cardio-inhibitory nerve apparatus. It is safe, then, to infer from this that atropine, in small doses, stimulates the heart muscle, and in large amounts it causes a peripheral paralysis of the cardio-inhibitory centres. In both cases a rapid pulse is produced. The decrease of the pulse-rate, under comparatively large doses, may similarly be attributed to the factors: stimulation of the cardio-inhibitory apparatus, and depression of the heart muscle.

### ON THE BLOOD-PRESSURE.

It is stated by high authorities that belladonna, or atropine, is a vaso-motor stimulant. Thus it is remarked: "As atropine does not augment the force of the individual cardiac beat, and as the increase in the number of the cardiac pulsations caused by it after section of the vagi is comparatively slight, it is exceedingly probable that the rise of the arterial pressure just spoken of, is due to contraction of the small vessels. This logical conclusion becomes almost a certainty when it is further known that after division of the cord, and consequent separation of the vessels from the vaso-motor centres, atropine is powerless to produce rise of the arterial pressure.' To this cumulative evidence must be added the experimental fact noted, that when a small dose of atropine is injected in the carotid artery—that is, into the vaso-motor centres—there is an instantaneous rise of blood-pressure."

I need not criticise snch statements, nor discuss the fallacy of such experimentation. But the fact is, that the action of atropine on the blood-pressure is by no means constant. The drug sometimes increases, and sometimes decreases the pressure in the normal animal. It appears, however, that when atropine causes a rise of the pressure, this increase is due to a stimulation of the centres in the medulla; and it has been proven by experimentation that the decrease is due mainly to depression of the vasomotor system, probably in both ways, peripherally and centrally.

*On the Respiration.*—Without sufficient proof, it seems to me, atropine has been looked upon for some time as essentially a respiratory stimulant. But the truth of the matter is, that here again we find the action of the drug to be uncertain, especially in normal animals. In those animals in which the vagi have been previously divided, the drug, it is true, almost always causes an inciease in the respiratory rate, and while this shows that the agent acts directly on the centers in the medulla, it does not prove that the drug is a respiratory stimulant. If this were so, we would have the same more or less constant effects in the normal animals, as is the case with cocaine, ammonia, caffeine, and strychnine, perhaps, and other drugs (and they are not many) which may be classed as true respiratory stimulants. Let us again refer to the paper of Reichert. In seven experiments performed on dogs, it was found that in five an increase in the number of respirations was effected some time during the observation, while in two it was practically unaffected. The increase took place sometimes immediately on the injection of the drug, at others not until several minutes after, while still in others there was, instead of an increase, either no effect at all or a tendency to a gradual decline. I have observed similar phenomena, and not until repeated experiments gave me almost invariably the same results, did I not make up my mind to discard atropine from the class of respiratory stimulants in the proper sense of the term. Thus, in conformity with such evidence, we must conclude that, as Reichert tersely puts it, "atropine acts upon the sespiratory function at the same time in two opposing ways: one (peripheral) tending to diminish, and the other (central) tending to increase, the increase or decrease of the respirations in the normal animal depending upon which one of these factors predominates."

From all these statements, based upon the results of experimentation, it is seen, then, that there is no true antagonism between opium and belladonna, in regard to their actions on the circulation or respiration. Each drug, as you may have observed, appears to influence the systems referred to in an opposing manner, but a close observation reveals the fact that the actions of each agent are inconstant and, therefore, unreliable. This being the case, there can not be between them a true antagonism. Indeed, there is a possibility that at some time or another the whole range of their actions, either on the circulation or the respiration, may run exactly the same course.

The antagonism of the two substances has been generally referred to their actions particularly on the respiratory function. Favorable results have been reported at various times in the journals, from the action of atropine in cases of human opium poisoning. These reports are comparatively few in number, and I am inclined to the belief that many of the facts embodied in these reports have been purely imaginary. In many of such cases of poisoning other measures have been employed as a rule, such as electricity, external heat, the administration of caffeine, and general stimulation, and there is no doubt in my mind that many of these cases have recovered in spite of atropine, while in some of those instances, if not all, in which a fatal issue has been observed, death has, undoubtedly, been hastened by atropine! I believe that the administration of atropine in cases of human opium poisoning is as fatal an error as the routine practice (a practice that is happily dying out, to the benefit of suffering humanity) of giving alcohol during ether or chloroform narcasis, which is the same as adding fuel to the fire!

In an elaborate research carried on by Prof. Wood and the writer, in the laboratories of the University of Pennsylvania, upon the subject of respiration, directed particularly to study the antagonistic actions of drugs on this function, special attention was paid to opium and belladonna. The results of this investigation will be published at an early date. It was precisely during this experimentation that I was able to notice the inconstant actions of both opium and belladonna when administered separately, and the failure to antagonize each other when given for this purpose. Morphine, as I intimated a few moments ago, sometimes produced an extraordinary increase in the number of the respiratory movements, accompanied by a corresponding increase in the amount of air going through the lungs. On the other hand, atropine sometimes not only did not cause an increase in the respiratory rate, but often failed to augment the depth of the breathing, and in such cases it actually diminished the amount of air passing through the lungs.

In regard to the influence of atropine in animals poisoned by morphine, the results were generally negative. While cocaine and strychnine, for instance, would nearly always restore promptly an animal fully under the toxic action of morphine, through respiratory stimulation, atropine was, under similar circumstances, powerless to do so, nay, this alkaloid would often hasten a fatal issue. This proves to my mind that atropine not only does not act as a respiratory stimulant, but it ought not to be looked upon as a physiological antidote to opium so far as the respiration, or the circulation, is concerned.

It has been said, and certainly with good reason, that no amount of experimentation can overthrow a clinical fact. Have we sufficient clinical evidence to prove the efficacy of atropine in human opium potsoning? I think not, and unbiased observations will bear me out. It cannot, however, be denied that there exists an antagonism between the two drugs in question, but such an antagonism must be looked for in other portions of the economy than in the circulation or the respiration.

The clinical observations of Sticker (*Centralbl. f. Med.*, March 26, 1892), in this respect, are particularly interesting. The author says that in certain cases of poisoning, "the antagonism of these drugs cannot be doubted, and that the want of general recognition of the fact is due to the few opportunities of observing it. Thus, the unpleasant effects of morphine used as a hypnotic may be prevented by the addition of atropine. In some cases morphine produces excitement, and if it is still necessary to use it, atropine will antagonize this. A subcutaneous injection of morphine lessens considerably the dilatation of the pupil produced by atropine drops, and an injection of morphine and atropine combined produces only a slight dilatation of the pupil. Irritation of the skin sometimes produced by morphine is prevented by atropine. The diaphoretic effects of morphine are sometimes troublesome; they do not occur if atropine be added. On the other hand, the dryness of the skin produced by atropine is remedied by morphine. One of the effects of morphine sometimes seen, and especially in those cases attended with early paralysis of the bladder, as in tabes, is retention of urine. Belladonna antagonizes it. Morphine mostly constipates; atropine has the opposite effect, especially in chronic constipation. In biliary and renal colic the two drugs should be combined, as not only is any obstruction to the passage of the stone lessened, but the power of propelling the stone is increased. In cases of heart disease with engorged pulmonary circulation, morphine is badly borne, whereas the addition of a small quantity of atropine does away with any disadvantages.''

I shall trespass upon your patience no longer. In concluding this imperfect paper, let me say, however, and insist upon it, that belladonna does not antagonize the action of opium on the respiration, or the circulation, and it seems to me that the ingestion of atropine in human opium poisoning is as unwarrantable and disastrous as the administration of alcohol in any shape or form in ether or chloroform narcosis.

---

DISCUSSION OF DR. CERNA'S PAPER, HELD BEFORE THE GALVESTON COUNTY MEDICAL SOCIETY, NOV. 21ST.

''Dr. Cerna's paper is an excellent one,'' said Dr. H. A. West. ''The importance of this subject is very great, for the impression exists generally among medical men that an antagonism between these two drugs does exist. I hope the discussion will be a full one.''

''Dr. Cerna's paper is so complete,'' remarked Dr. Randall; ''his experimental study of the physiological action of atropine and opium has been so thorough, that there remains little or nothing to be discussed. In the treatment of opium poisoning, after the drug has been gotten rid of by a suitable emetic, there remains but two indications to be met, viz.: to maintain respiration and to keep the circulation from failing. The doctor shows that atropine subserves neither of these purposes. It is taught pretty generally that atropine is the most reliable respiratory stimulant that we have; but reviewing a number of cases of poisoning by opium in which atropine has been used, I have never been able to see that this drug sustains its high reputation; in fact, I have never seen any chemical evidence that it is a respiratory stimulant. Dr. Cerna shows that there is no experimental evidence for such a belief. With due deference to the authorities, I have taught that atropine might be used in opium poisoning, but that it must play a secondary part to strychnine and caffeine which are true respiratory stimulants. Inherited dogmas can't be accepted as facts without undergoing the crucial tests of modern science, and gradually the wheat is being separated from the chaff. Such good work done by Dr. Cerna and Dr. Reichert on this subject cannot be too highly praised. There is one point the doctor did not mention, possibly because he thought it was too well understood, and that is the apparent antagonism of the two drugs on the pupil. I say apparent, for the antagonism is not real. Opium produces myosis by a centric stimulation of the oculo-motor nerves and perhaps by depressing the sympathetic; whereas atropine produces mydriasis by stimulating peripherally the sympathetic nerve fibres of the iris and paralyzing the oculo-motor fibres."

"It has been my fortune," said Dr. J. E. Thompson, "to have passed a considerable portion of my professional life in a large hospital, where I have met with a number of cases of severe opium poisoning, and I have thought that on several occasions some stimulant effect on the circulation had resulted from the administration of atropine in such cases. I recall one case in particular, where the patient had taken two grains of morphine in the course of half an hour, with the result of producing a profound comatose condition. The electric battery, wet towels, hot coffee were all tried, and the patient was then given $\frac{1}{20}$ gr. of atropine subcutaneously and in twenty minutes an improvement was noted, which I had thought up to the time of hearing Dr. Cerna's paper, was due to the atropine; but now I am in doubt about it. Perhaps the recovery was brought about by the other agents used. Another patient poisoned with opium to whom atropine was given, was brought around at the time, but intense vomiting followed, and in three days the patient died. Possibly the death here was caused by atropine."

"While looking up this subject," said Dr. W. Keiller, "in the authorities at my command, I have been struck with the great confusion of ideas and statements regarding it. I think that atropine lessens the tendency to vomiting produced by the latter drug. I would give digitalin, strychnine and ammonia in a case of laudanum poisoning. I would like to ask Dr. Cerna whether, in his opinion, apomorphia might be given as an antagonist. I don't think it is a marked depressant, and it is a powerful respiratory stimulant. If administered in opium poisoning, even if emesis does not follow, still its effect on the respiratory centre would be beneficial. In the production of chloroform anæsthesia, I have found it of benefit, to administer about five minutes before the anæsthetic, about one-third of a grain of morphine combined with some atropine, the effect being to produce a calming influence and a kind of resignation of the patient to the anæsthetic. Perhaps the action of the atropine on the terminations of the vagus is such as to lessen the tendency to syncope, which is generally looked upon as a reflex tendency in the early stage of anæsthesia."

"Will Dr. Cerna," asked Dr. Sampson, "give us the treatment for opinm poisoning? In my opinion the use of apomorphia wonld aggravate the trouble. I think we should consider the possibility of some opium having passed the stomach into the intestines in cases of poisoning, and although I have never given drastics, still I think something should be done to get rid of it."

"I repeat," replied Dr. Cerna, "that there is no evidence of any kind to show that belladonna is of service in the treatment of opium poisoning, for in all the reported cases other agents besides the belladonna have been used. The evidence is that apomorphia is a powerful depressant and would therefore be not indicated in opium poisoning, because we wish always to stimulate the patient, not to depress. I think the administration of morphine before chloroform or ether anæsthesia is good, because it tends to enhance the production of anæsthesia. As respiratory stimulants, strychnine, cocaine, and caffeine head the list, while as a cardiac stimulant under such circumstances there is nothing better than digitalis. I have seen the hearts of animals apparently perfectly dead, revive in the course of a few seconds after the administration of digitalis. Ammonia is a very diffusible stimulant and its action is too rapid. The proper treatment of opium poisoning is stimulation of all kinds; but sometimes all the agents mentioned fail of effect and then the best thing is artificial respiration. I have seen animals thrown aside in the laboratory as dead, from the action of chloroform for instance, revived by artificial respiration."

"I wish to mention a case," said Dr. Flavin, "that I saw in the hospital during the summer. When I saw the case, respirations were following each other at the rate of four per minute. Electricity was then applied to the phrenics, and instantly the natural respirations ceased; but by artificial respiration the patient was kept alive for 6 or 7 hours; the patient during all this time making not a single voluntary respiratory movement."

Dr. Randall remarked that this case was not one of opium poisoning, but one of some trouble with the respiratory centre.

"My faith has for some years past," said Dr. Truehart, "been placed in artificial respiration and cardiac stimulation and *not* in atropine in the treatment of opium poisoning."

"Chloroform kills by its action upon the heart itself," remarked Dr. Cerna, "and atropine is not indicated under such circumstances, because it paralyzes the heart muscle, not stimulating it."

